# Draft Genome Sequence of the *Gluconobacter oxydans* Strain DSM 2003, an Important Biocatalyst for Industrial Use

**DOI:** 10.1128/genomeA.00417-14

**Published:** 2014-05-01

**Authors:** Binbin Sheng, Jun Ni, Chao Gao, Cuiqing Ma, Ping Xu

**Affiliations:** a State Key Laboratory of Microbial Technology, Shandong University, Jinan, China; b State Key Laboratory of Microbial Metabolism & School of Life Sciences and Biotechnology, Shanghai Jiao Tong University, Shanghai, China

## Abstract

*Gluconobacter oxydans* strain DSM 2003 can efficiently produce some industrially important building blocks, such as (*R*)-lactic acid and (*R*)-2-hydroxybutyric acid. Here, we present a 2.94-Mb assembly of its genome sequence, which might provide further insights into the molecular mechanism of its biocatalysis in order to further improve its biotechnological applications.

## GENOME ANNOUNCEMENT

*Gluconobacter oxydans*, an obligate aerobic, Gram-negative, and rod-shaped acidophilic organism that belongs to the family *Acetobacteriaceae*, is known for its incomplete oxidation of a wide range of carbohydrates and alcohols (1). The corresponding products (aldehyde, ketone, and organic acid) are excreted almost completely into the medium. *G. oxydans* strains can be used industrially to produce l-sorbose from d-sorbitol; d-gluconic acid, 5-ketogluconic acid, and 2-ketogluconic acid from d-glucose; dihydroxyacetone from glycerol; and 6-amino-l-sorbose from 1-amino-d-sorbitol for the synthesis of Miglitol (1–3). The complete genome sequence of *G. oxydans* 621 H (DSM 2343) is publicly available (3). The genome data provide useful information for metabolic reconstruction of the pathways leading to some industrially important products derived from alcohols and sugars.

*G. oxydans* can also be employed for the enantioselective oxidation of different racemic primary alcohols for the production of enantiomerically pure carboxylic acids (2). In previous studies, *G. oxydans* DSM 2003 efficiently catalyzed racemic 1,2-propanediol and racemic 1,2-butanediol into (*R*)-lactic acid and (*R*)-2-hydroxybutyric acid, respectively (4, 5). (*R*)-Lactic acid and (*R*)-2-hydroxybutyric acid are important building blocks for the production of 2-oxo-carboxylates, biodegradable material, glycols, halo esters, and epoxides (6–9). These compounds are important intermediates of pharmaceuticals (9–11). To better understand the biocatalytic process of *G. oxydans* DSM 2003 and to further improve its biotechnological applications, we sequenced the genome of the strain.

The draft genome sequence of *G. oxydans* DSM 2003 was obtained using the Illumina GA system; sequencing was performed by the Chinese National Human Genome Center at Shanghai, China, with a paired-end library. The reads were assembled by using the Velvet software (12). The genome was annotated using the Rapid Annotations using Subsystems Technology (RAST) automated annotation server (13). The G+C content was calculated using the genome sequence. The functional description was determined by using Clusters of Orthologous Genes (14). The rRNA and tRNA genes were identified by RNAmmer 1.2 (15) and tRNAscan-SE (16), respectively.

The draft genome sequence of DSM 2003 has a G+C content of 60.9%. The number of contigs (>100 bp) was 147, and the number of bases was 2,938,182. There are 2,792 putative coding sequences (CDS) (935 bp average length), 50 tRNA genes, and 2 rRNA operons in the genome sequence. The coding percentage is 67.8%, and 1,892 CDS have predicted functions.

There are 363 subsystems represented in the draft genome sequence. The genes encoding proteins responsible for enantioselective oxidation of racemic 1,2-propanediol for the production of (*R*)-lactic acid were successfully annotated. Several annotated NAD-independent lactate dehydrogenase genes, which might play important roles in 2-hydroxy-carboxylate oxidation (17–19), were also annotated. The obtained genome sequence provides useful hints for strain improvement; for example, the annotated NAD-independent lactate dehydrogenase genes provide targets for gene knockout to further improve the yield of final (*R*)-lactic acid and (*R*)-2-hydroxybutyric acid production by *G. oxydans* DSM 2003*.*

### Nucleotide sequence accession numbers.

This whole-genome shotgun project has been deposited at DDBJ/EMBL/GenBank under the accession no. AYTY00000000. The version described in this paper is the first version, with accession no. AYTY01000000.
